# Prevalence of Vitamin D Deficiency in a Young Adult With Acute Spinal Cord Injury

**DOI:** 10.7759/cureus.13791

**Published:** 2021-03-09

**Authors:** Shah Waliullah, Deepak Kumar, Dharmendra Kumar, Prakash G Tewari, Vineet Kumar, Rajeshwar N Srivastava

**Affiliations:** 1 Department of Orthopaedic Surgery, King George's Medical University, Lucknow, IND; 2 Department of Orthopaedic Surgery, Dr. Ram Manohar Lohia Institute of Medical Sciences, Lucknow, IND

**Keywords:** vitamin-d deficiency, acute spinal cord injury, osteoporosis

## Abstract

Introduction: Vitamin D deficiency has been implicated as an etiologic factor responsible for osteoporosis and various skeletal and extra-skeletal issues in spinal cord injury patients. There is a dearth of publications regarding the prevalence of vitamin D deficiency in acute spinal cord injury (ASCI) patients, thus it becomes imperative to study the status of vitamin D in ASCI cases to make an early diagnosis and start treatment for osteoporosis. Apart from this, we also planned to evaluate other factors associated with vitamin D deficiency in our subset of patients.

Material and methods: This cross-sectional cohort study included patients with acute thoracolumbar spinal cord injury patients admitted to a tertiary trauma centre between July 2019 and July 2020. Patients were assessed clinically and classified as per the American Spinal Cord Injury Association (ASIA) scale. Demographic details along with the mode of trauma and duration of injury were noted. Serum 25(OH) vitamin D3 levels were measured by chemiluminescence immunoassay. Depending upon serum 25(OH) vitamin D3 level, patients were classified into vitamin D deficient with serum level less than 20 ng/ml, vitamin D insufficient with serum level between 21-29 ng/ml and vitamin D sufficient with serum level greater than 30 ng/ml.

Results: Mean vitamin D level in 85 ASCI subjects (mean age 30.82 ± 6.77 years, 60 males) was 20.56 ± 11.22 ng/ml. Fifty subjects (58.82%) were vitamin D deficient, 15 subjects (17.64%) were vitamin D insufficient and the rest (n=20, 23.52%) were vitamin D sufficient. There was no significant difference in vitamin D levels as per gender, age, mode of trauma, type of injury and injury location. Patients admitted on the fifth day of injury had maximum vitamin D levels (mean 25.7143 ± 8.32 ng/ml), but it was also insignificant. The mean vitamin D level of subjects with samples taken during the summer season was significantly higher as compared to the winter season (p value <.05).

Conclusion: Vitamin D deficiency is widely prevalent in ASCI patients at admission to the trauma centre. Seventy-six percent of patients had vitamin D levels below 30 ng/ml in our study. Routine measurement of 25(OH) vitamin D3 levels at the time of admission is recommended for early diagnosis of vitamin D deficiency. Early treatment will be helpful in the prevention of osteoporosis and its long-term related consequences.

## Introduction

Vitamin D is a steroid hormone and is synthesized in the skin or ingested through diet and converted into the active form after two hydroxylations, firstly at the liver and then the kidney. Its active metabolite binds to the nuclear vitamin D receptor (VDR) and carries out various genomic activities. Vitamin D has been associated with modifying the expression of several genes associated with axogenesis, myelination, neuronal-cell differentiation and repair [1,2].

Spinal cord trauma is a devastating injury, and its sequelae range from normal neurology to complete paralysis and even death. Patients with normal neurology gain ambulation in due time depending upon injury severity; however, patients with incomplete and complete spine injury face prolonged immobilization and several problems pertaining to immobilization. Because of immobilization and disturbed endocrinal axis, osteoporosis and consequent fractures are challenging threats to manage in these patients. Vitamin D deficiency has been implicated as an etiologic factor responsible for osteoporosis and various skeletal and extra-skeletal issues in spinal cord injury patients [3-7].

Several reports have highlighted the presence of vitamin D deficiency in chronic spinal cord injury patients [5-7]. There are very few reports regarding the prevalence of vitamin D deficiency in acute spinal cord injury (ASCI) patients, thus it becomes imperative to study the status of vitamin D in ASCI as it will help to know whether spinal trauma is an initiating factor for vitamin D deficiency, or if they are previously vitamin D deficient before the trauma [8,9]. Moreover, it will help to start early treatment in deficient cases to prevent future problems. With this study, we also want to evaluate various factors associated with vitamin D deficiency in our region's spinal cord injury patients.

## Materials and methods

This cross-sectional cohort study included acute thoracolumbar spinal cord injury patients admitted to our tertiary trauma centre from July 2019 to July 2020. Institutional ethical clearance (IEC number: 95th ECM II A/P5) was taken before the initiation of the study. The data was retrieved from the online records of the institutional database. Data of all the cases admitted between the said period suffering from traumatic SCI within a week of injury and satisfying our inclusion and exclusion criteria were selected for the study. All the cases of traumatic thoraco-lumbar (D1-L5) spine injury presenting to us within one week of injury were recruited in the study. The data was recorded for radiological and clinical findings. The clinical findings were recorded as per the American Spinal Cord Injury Association (ASIA) scale [10] and divided into three categories: ASIA A complete injury, ASIA B, ASIA C, ASIA D as incomplete injury and ASIA E as neurologically intact cases. Demographic details, along with the mode of trauma and duration of injury, were noted. Patients with injury more than seven days were excluded. Any patient with previous treatment for vitamin D deficiency or taking medications affecting vitamin D metabolism or suffering from malignancy, endocrinal disorder, hepatic disorder, renal disorder, or a gastrointestinal disorder affecting calcium and vitamin D metabolism, were excluded from the study. Serum 25(OH) vitamin D3 levels as measured by chemiluminescence immunoassay were recorded. Vitamin D levels from the samples taken at the time of admission and the season during which the sample was taken were also documented; whether summer (March to August) or winter (September to February). Depending upon serum 25(OH) vitamin D3 levels, the patients were classified as vitamin D deficient (serum level less than 20 ng/ml), vitamin D insufficient (serum level between 21-29 ng/ml) and vitamin D sufficient (serum level greater than 30 ng/ml) [11].

Statistical analysis

All statistical analyses were performed by Statistical Package for Social Sciences (SPSS) version 16.0 (SPSS Inc., Chicago, IL, USA) and Graph Pad Prism version 5 (GraphPad Software, La Jolla, CA, USA). The quantitative data were analyzed using the Mann Whitney U test/t-test, and Kruskal Wallis/analysis of variance (ANOVA) was used to compare the different groups of variables. P value <0.05 was considered to be statistically significant. 

## Results

**Figure 1:**
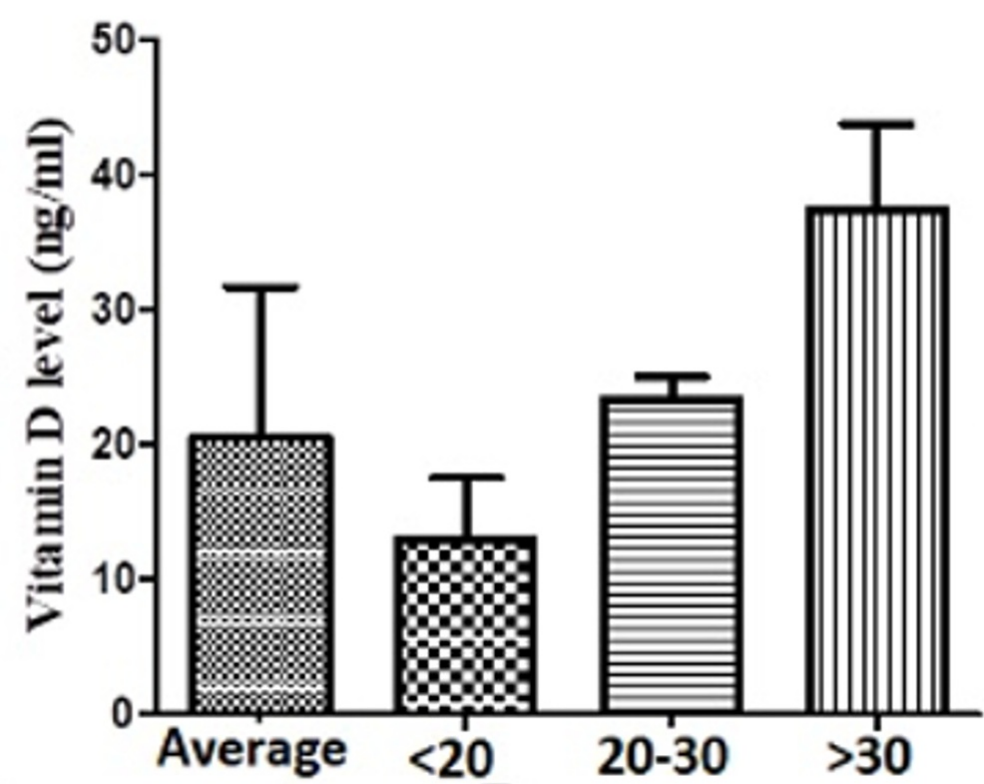
Vitamin D levels in the study population

Mean vitamin D level in 85 ASCI subjects (mean age 30.82 ± 6.77 years, 60 males) was 20.56 ± 11.22 ng/ml. Fifty subjects (58.82%) were vitamin D deficient, 15 subjects (17.64%) were vitamin D insufficient and the remaining 20 subjects (23.52%) were vitamin D sufficient (Figure 1, Table 1). 

**Table 1:** Vitamin D levels in the study population

	Vitamin D levels (n=85) (in ng/ml)	<20 (n=50) (in ng/ml)	21-30 (n=15) (in ng/ml)	>30 (n=20) (in ng/ml)
Mean	20.56	12.95	23.35	37.48
Std. Deviation	11.22	4.62	1.68	6.30

There were no significant differences in vitamin D levels as per gender, age distribution, mode of trauma or native origin (Table 2). There was no association between the type of injury and the level of injury (Table 2). 

**Table 2:** Demographic and clinical characteristics of patients ASIA: American Spinal Cord Injury Association, MVA: Motor Vehicular Accident

		25(OH) Vitamin D levels ng/ml		
	N(%)	Mean	Std. Deviation	P value
Sex				
Male	60(70.51	31.13	6.74	0.31
Female	25 (29.41)	30.08	6.93	
Mode of Trauma				
Fall from height	41(48.24)	20.08	12.03	0.35
MVA	34(40.00)	21.94	10.91	
Others	10(11.76)	17.83	8.93	
Native Area				
Rural	46(54.12)	19.78	10.39	0.64
Urban	39(45.88)	21.47	12.21	
Injury Type				
ASIA-A	28(32.94)	21.16	11.30	0.77
ASIA-B/C/D	33(38.82)	19.57	11.63	
ASIA-E	24(28.24)	21.22	10.94	
Level of Injury				
Dorsal	16(18.82)	19.74	10.41	0.86
Dorso-Lumbar	52(61.18)	20.80	11.20	
Lumbar	17(20.00)	20.59	12.62	

Vitamin D level on the fifth day of injury was maximum (mean 25.7143 ± 8.32 ng/ml), but it was also insignificant (Table 3). 

**Table 3:** Vitamin D levels in subjects with respect to duration of injury

Duration of injury (in days)	Vitamin D levels (in ng/ml)		P value
	Mean	Std. Deviation	
1	19.5179	10.10567	0.27
2	22.4440	12.31114	
3	21.8929	12.58763	
4	13.1000	11.72035	
5	25.7143	8.32275	
6	16.1333	5.27289	
7	13.2000	.	

**Figure 2:**
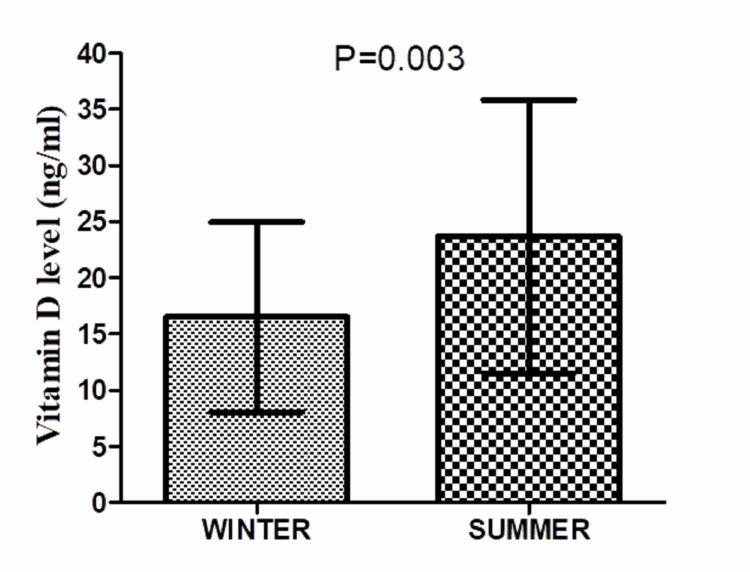
Vitamin D level variation with season

Mean vitamin D level of subjects with samples taken during the summer season was 23.67 ± 12.15 ng/ml. It was higher and significant compared to mean vitamin D level of subjects with samples taken during the winter season (16.5189 ± 8.45 ng/ml) (Table 4, Figure 2).

**Table 4:** Vitamin D levels in subjects with relation to the seasonal presentation

Season	Vitamin D levels (in ng/ml)		P-value
	Mean	Std. Deviation	
WINTER	16.5189	8.45290	0.003
SUMMER	23.6708	12.15298	

## Discussion

This study describes the prevalence of vitamin D deficiency in ASCI patients presenting within a week of the injury. We found that in our study population almost 76.5% (65/85) cases had either insufficient levels or deficient levels of serum vitamin D. The observed low level of 25(OH) vitamin D3 may also be due to acute systemic inflammatory response to spinal trauma that can result in a decrease in vitamin D binding protein and subsequently decreases 25(OH) vitamin D3 levels [10]. However, we did not observe any significant association between vitamin D levels and the duration of the injury. Future studies with serial measurement of vitamin D binding protein and 25(OH) vitamin D3 level in cases of ASCI will probably answer this question and help us to better understand the exact cause of vitamin D deficiency in this cohort.

In our study, 76.5% of patients had vitamin D levels below 30 ng/ml and the mean 25(OH) vitamin D3 level was 20.56 ± 11.22 ng/ml. Nemunaitis et al. [11] observed a 93% prevalence of vitamin D deficiency in his study on 100 spinal cord injury patients, which included acute, subacute and chronic SCI patients with a mean 25(OH) vitamin D3 level of 16.29 ± 7.73 ng/ml. Further, in their study, there were 45 patients with a duration of injury less than one month and they had a mean serum level of 14.5 ± 7.0 ng/ml.

We did not find any significant relation in vitamin D levels amongst the cases presenting to us on the day of the injury and those presenting on the seventh day after the injury, nor did we find any correlation between demographic details, mode of trauma, level of injury, or neurological status in our cases. Nemunaitis et al. [11] had similar observations on these criteria in their study.

In our study, seasonal variations were found to have an influence on vitamin D levels. The patients who were admitted in the summer season had a higher level of vitamin D as compared to those admitted in the winter season. Oleson et al. [12] found that 25(OH) vitamin D3 levels were less than 32 ng/mL in 65% of ASCI patients who present with injury in the summer season, whereas this figure was 84% in cases of ASCI presenting in the winter season. They observed a mean 25(OH) vitamin D3 level of 28.03 ± 15.25 ng/ml in ASCI during the summer season and 21.72 ± 12.56 ng/ml in ASCI winter season. In our series, vitamin D level was 23.67 ± 12.15 ng/ml in cases of ASCI admitted during the summer season and 16.51 ± 8.45 ng/ml during the winter season.

Our study was done in patients belonging to the northern part of the country with 26º latitude. In winters, sunlight is not optimal to synthesize vitamin D. We observed a low level of vitamin D in the winter in our subjects, correlating with other previous studies [13,14] done at the same geographical area in a different cohort of patients. Our study is probably one of the first to report the prevalence of vitamin D deficiency in ASCI at the time of admission in our region.

In one of the animal studies available in the literature, supplementing vitamin D in the setting of ASCI with neurological deficit within a week of injury was associated with neurological recovery [15]. We also had 76.5% of cases in our study with vitamin D deficiency, thus an early diagnosis and supplementation with vitamin D might also be helpful for neurological recovery in such cases. A detailed study in the future might provide us with a better insight into this aspect of treatment.

The major limitation of our study is that it was not possible for us to ascertain the prior status of vitamin D levels in cases presenting to us with SCI. In any case, decreased levels of vitamin D if diagnosed in time, especially in such cases, will definitely help in decreasing complications and managing these cases more efficiently. Another limitation is that we did not enquire about the dietary details of patients and sun exposure that may impact the analysis and give insight regarding vitamin D status before the injury. We did not take ethnic and racial factors into account in our study, which have been reported as significant in previous studies [11,12]. A prospective study with a larger sample size and evaluation of vitamin D levels over a longer period of time post-injury and correlating it with the neurological recovery pattern will help us gain further information which could guide us for future research plans. Multi-centre studies with a larger sample size are required to elaborate on the epidemiology of vitamin D deficiency in ASCI patients. We took a cut-off value of 20 ng/ml as a deficiency state and between 21-30 ng/ml as an insufficiency state [9]; however, these levels are variable in the literature [5,7] and they vary according to the race and region as well. Thus, to have standard cut-off values with respect to the population being studied becomes important to diagnose and classify vitamin D deficiency status in ASCI cases. Further studies can be undertaken to generate robust data to formulate guidelines regarding diagnostic cut-off values of 25(OH) vitamin D3 level in ASCI patients.

## Conclusions

Vitamin D deficiency was significantly prevalent in ASCI patients admitted to our hospital. 76.5% of patients had vitamin D levels below 30 ng/ml in our study. Routine measurement of 25(OH) vitamin D3 levels at the time of admission is recommended for early diagnosis of vitamin D deficiency. Early treatment will be helpful in the prevention of osteoporosis and its related consequences in the long term. Future studies are warranted to investigate the role of early vitamin D supplementation in neurological recovery from spinal cord injury.
